# Application of vibrational spectroscopies as process analytical techniques for monitoring fermentation and the conversion of lignocellulosic biomass by oleaginous filamentous fungi

**DOI:** 10.1186/s12934-025-02868-w

**Published:** 2025-12-09

**Authors:** Simona Dzurendova, Cristian Bolaño Losada, Benjamin Xavier Dupuy-Galet, Ondrej Slany, Kai Fjaer, Francesca Di Bartolomeo, Sidsel Markussen, Alexander Wentzel, Anikó Várnai, Line Degn Hansen, Svein Jarle Horn, Achim Kohler, Volha Shapaval, Boris Zimmermann

**Affiliations:** 1 Faculty of Science and Technology, Norwegian University of Life Sciences, Postbox 5003, Ås, 1432 Norway; 2 Faculty of Chemistry, Brno University of Technology, Purkyňova 464/118, Brno, 61200 Czech Republic; 3 SINTEF Industry, Postbox 4760, TorgardenTrondheim, 7465 Norway; 4 Faculty of Chemistry, Biotechnology and Food Science, Norwegian University of Life Sciences, Postbox 5003, Ås, 1432 Norway

**Keywords:** Oleaginous microorganisms, Biodiesel, Lignocellulose, Fourier transform infrared spectroscopy, Fourier transform raman spectroscopy, FTIR microscopy, Mucor, Bioprocess monitoring, Simultaneous saccharification and fermentation

## Abstract

**Background:**

Oleaginous filamentous fungi, such as *Mucor circinelloides*, are capable of accumulating high levels of single cell oil (SCO), making them attractive candidates for the production of biodiesel and other oleochemicals. Lignocellulosic feedstocks offer an abundant and cost-effective carbon source for SCO production due to their high polysaccharide content. However, most oleaginous microorganisms cannot directly utilize cellulose and hemicellulose polysaccharides, necessitating their conversion into monosaccharides. Lignocellulosic substrates can be saccharified either separately from fermentation (separate hydrolysis and fermentation; SHF) or simultaneously (simultaneous saccharification and fermentation; SSF). This study evaluated SSF using *M. circinelloides*, as well as SHF cultivations on two types of lignocellulosic hydrolysates, and two control fermentations, with process monitoring via four vibrational spectroscopy techniques: Fourier Transform Infrared (FTIR) spectrometer with fibre optic probe, FTIR microspectrometer, FTIR spectrometer with high throughput setting (HTS), and FT-Raman spectrometer with HTS.

**Results:**

Quantitative estimation of glucose in the cultivation media and lipid content in the biomass was achieved using PLSR analysis of FT-Raman measurements from the cell suspension. FT-Raman spectroscopy demonstrated exceptional capability for online and at-line process monitoring among the tested techniques. It enabled direct and rapid analysis of raw cell suspensions (containing growth media, cellulose-rich pulp substrate, and fungal biomass) without the need for sample pre-treatment, purification, or modification. FT-Raman provided comprehensive biochemical profiles, effectively detecting key chemical changes in both the cellulose-rich pulp substrates and the fungal biomass, including lipid accumulation by the oleaginous fungi. FTIR with fiber optics is effective for monitoring glucose in SHF processes, but its accuracy is limited in SSF processes due to the very low glucose concentrations. The study demonstrates that FTIR microspectroscopy is a valuable tool for lab-scale fermentation process development, as well as for investigating the bioconversion of lignocellulosic biomass into fungal biomass and metabolites.

**Conclusions:**

FT-Raman spectroscopy is highlighted as a powerful process analytical technology (PAT) tool for real-time or near-real-time monitoring of SSF processes for intracellular SCO production. Its ability to provide rich chemical information rapidly and without extensive sample preparation holds significant promise for optimizing industrial SCO production from lignocellulosic feedstocks.

**Supplementary Information:**

The online version contains supplementary material available at 10.1186/s12934-025-02868-w.

## Introduction

Oleaginous filamentous fungi can produce microbial single cell oil (SCO) with diverse biochemical and physicochemical properties, and thus can serve as a source of oleochemicals [1, 2]. Under carbon-rich and nitrogen-limited growth conditions, oleaginous filamentous fungi can accumulate a high amount of lipids, reaching 70–85% of their dry weight [3]. Moreover, they have versatile metabolism that enables fermentation processes on a wide range of feedstocks, such as waste and rest biomass [4–7]. Due to these favourable traits, fungal oils are considered a viable alternative to plant and animal oils for production of food, feed and nutraceuticals [2, 8]. Furthermore, fungal biorefineries could serve as an alternative to petrochemical industry as a sustainable source of platform chemicals and fuels [2, 6, 9]. For example, oleaginous Mucoromycota fungi can co-produce lipids, biopolymers (chitin, chitosan and polyphosphates), and carotenoid pigments, all in a single cultivation process [3, 10–13]. However, economic sustainability of SCO production by oleaginous filamentous fungi requires low-cost nutrient feedstocks and cost-effective biotechnological processes [6].

Due to their wide availability and high polysaccharide content, lignocellulosic feedstocks are regarded as the best low-cost carbon sources for SCO production [6, 14]. However, lignocellulosic biomass usually needs to be converted into monosaccharides since the majority of oleaginous microorganisms are not able to directly utilise cellulose and hemicellulose polysaccharides. Pretreatment is often required to deconstruct the complex matrix of polymers (cellulose, hemicellulose and lignin) and make the polysaccharides available for a subsequent enzymatic hydrolysis. One approach envisions separate hydrolysis and fermentation (SHF), where in the first step pretreated lignocellulosic biomass is hydrolysed into monosaccharides, and in the second step these monosaccharides are further converted into SCOs by oleaginous filamentous fungi. These steps can be carried out sequentially in the same vessel by adjusting the conditions between processes, or alternatively, in different vessels. The other approach is based on simultaneous saccharification and fermentation (SSF), where lignocellulose hydrolysis (saccharification) is integrated with fermentation into one simplified and cost-effective process. Despite its advantages, it entails more complexity to monitoring the bioprocess progression since the biomass consist of a fungal and lignocellulose mixture. In general, SSF processes using Mucoromycota fungi are scarce and commonly applied for ethanol production from lignocellulosic biomass, while their utilisation in SCO production is still under development [15, 16].

The main challenge in the bioprocess industry is reducing process variability caused by variability of raw materials, variability introduced by process instrumentation and handling, and variability generated during cellular reproduction and growth [17]. Since a typical fermentation process is afflicted by all these types of variability, it is considered the most complex individual process within biotech manufacturing. As such, it has the greatest influence on the manufacturing output and product quality. Thus, timely monitoring and control of fermentation is a crucial requirement for establishing economically viable bioprocesses by providing early identification of shortcomings and disruptions, and implementation of appropriate corrections. Unfortunately, a typical bioprocess is usually monitored by a handful of process parameters, such as pH, conductivity, temperature, and dissolved gases. Although these parameters are important, process operators need critical process parameters in order to detect sources of variability and improve process performance. Critical process parameters, such as biomass, product, and substrate concentrations and compositions, are rarely available by at-line, on-line (bypass) and in-line (in-situ) sensors. Thus, modern spectroscopy-based sensors combined with advanced data analytics are emerging as a solution for obtaining detailed chemical information for bioprocess monitoring, control and optimization [18–21].

Vibrational spectroscopy covers a number of infrared and Raman spectroscopy techniques for fast and inexpensive measurements of various types of samples and processes [22, 23]. These techniques are ideal process analytical technology (PAT) tools since they provide comprehensive chemical information due to simultaneous measurement of the molecular constituents in the process via detection of their specific molecular vibrations and implementation of chemometrics and data analysis methods [24–29]. Both Fourier transform infrared (FTIR) and Raman spectroscopies are suitable for monitoring growth media and various types of microbial products, such as lipids, alcohols, and pigments [26, 28, 30–32]. Moreover, both methods offer versatile measurement setups, from in-situ monitoring in bioreactors by fibre-optic probes [25, 33–36], high-throughput at-line screening [2, 24, 37], to the detailed cellular imaging by microspectroscopies [38, 39]. In our previous studies, we have demonstrated that FTIR and FT-Raman spectroscopies can be used for chemical characterization of Mucoromycota fungi by measuring biomass and growth media with high-throughput screening (HTS) units, attenuated total reflection (ATR) units, and microspectroscopies [2, 10, 11, 24, 37, 39–44]. Moreover, we have recently demonstrated that FT-Raman spectroscopy can be used as ultimate PAT tools for simultaneous in-situ real-time measurements of multiple critical process parameters during microbial lipid production, such as concentration of nutrients, microbial biomass (yeasts and thraustochytrids), and intracellular SCOs and carotenoids [27]. Finally, we have demonstrated that vibrational super-resolved microspectroscopy can assess phenotypic heterogeneity of the microbial population in the lipid-production fermentation at the single-cell level [45].

Monitoring SSF process for SCO production presents unique challenges due to the complexity of the process. The presence of a large amount of starting lignocellulosic biomass makes it difficult to accurately track changes in substrate composition over time, especially with standard methods such as optical density sensors. These sensors are commonly used to monitor microbial growth but are unable to distinguish between suspended lignocellulosic particles and microbial cells, leading to inaccurate measurements. The high concentration of solid biomass in the system interferes with light scattering and absorption, further complicating the use of these traditional techniques for process monitoring. Additionally, differentiating between microbial biomass and the residual lignocellulosic material is particularly challenging, as their chemical and structural signatures often overlap. Furthermore, measuring intracellular metabolites, such as lipids, within the microbial cells adds another layer of difficulty, especially in the context of a heterogeneous system where microbial cells are embedded within the lignocellulosic matrix. These challenges highlight the need for advanced analytical tools capable of providing detailed and accurate insights into such complex fermentation systems.

In this study, we have assessed the potential of FTIR and FT-Raman spectroscopy techniques for monitoring of SHF and SSF processes employing *Mucor circinelloides* filamentous fungus. Thus, the novelty of the present study lies in both, first-time assessment of (near)-real-time spectroscopy monitoring of bioprocess employing filamentous fungus and a novel SSF and SHF processes based on lignocellulosic substrates. *Mucor circinelloides* is a known producer of SCOs, and it was the first specie utilised in commercial production of microbial oil rich in γ-linolenic acid [46]. In addition to production of SCOs, *Mucor circinelloides* has potential to serve as microbial cell factory for carotenoid pigments as well as biopolymers, such as chitin, chitosan and polyphosphates [3, 10, 11]. We have conducted SSF with *M. circinelloides*, as well as SHF cultivations on two different types of lignocellulosic hydrolysates and two different types of control fermentations. Cultivations were analysed using four different spectroscopic techniques: an FT-Raman spectrometer with a high-throughput setting, an FTIR spectrometer with a fibre optic probe, an FTIR microspectrometer, an FTIR spectrometer with a high-throughput setting. The spectroscopic measurements were conducted on three different types of samples: cell suspension (i.e., unrefined whole bioreactor content comprising growth media and biomass), culture supernatant, and dry biomass. The application of these techniques was simulated in both on-line and at-line settings to evaluate their potential for real-time and near-real-time monitoring of the fermentation process. A schematic illustration of the overall research methodology is presented in the Supplementary Material (Figure S1).

## Materials and methods

### Fungal strains and preparation of inoculum

Mucoromycota oleaginous filamentous fungus *Mucor circinelloides* VI 04473 was obtained from the Norwegian School of Veterinary Science, Oslo, Norway (VI). In our previous studies, the selected strain exhibited compatible traits with SSF [47], as well as good potential for lipid, chitin, chitosan. and polyphosphate production [2, 3, 10, 11].

For the preparation of fresh spore suspension, *Mucor circinelloides* was cultivated on malt extract agar (MEA). MEA plates were prepared by dissolving 30 g of MEA (Merck, Germany) in 1 L of distilled water and autoclaved at 121 °C for 15 min. The agar plate cultivation was performed for 5 days at 25 °C. Fresh spores were collected by washing the mycelium with 0.9% NaCl sterile solution and used for the preparation of pre-cultures. The pre-culture medium was composed of 40 g/L glucose and 10 g/L yeast extract. Each 500 mL Erlenmeyer flask contained 150 mL of the pre-culture medium and was inoculated with 5·10^5^ spores/mL. Pre-cultures were grown at 25 °C and 150 rpm for 2 days. Each bioreactor was inoculated with 150 mL (10%) of pre-culture.

### Lignocellulose pretreatment, cellulose-rich pulp and hydrolysates

The substrates used as the carbon source in this study where derived from Norway spruce (*Picea abies*) biomass pretreated by the BALI™ process of the biorefinery Borregaard (Sarpsborg, Norway) [48]. The BALI™ process entails a sulfite-pulping pretreatment that results in two main streams: (1) an insoluble part consisting of a cellulose-rich BALI™ pulp (87.4% glucan content), and (2) a soluble part consisting of lignosulfonates and hemicellulose sugars. The Excello 90 is the commercial enzymatic hydrolysate of BALI™ pulp produced by Borregaard. It is a mixture of monomeric sugars, with glucose as the main component (approx. 80% of total carbohydrates), and xylose, mannose, galactose, fructose, and arabinose as minor components [48, 49]. The NMBU BALI™ lignocellulosic hydrolysate was prepared in-house using BALI™ pulp and enzymatic saccharification by Cellic CTec2^®^ (Novozymes A/S, Bagsværd, Denmark), as described in [50]. The BALI™ pulp was used as is in combination with Cellic CTec3^®^ (Novozymes A/S, Bagsværd, Denmark) for the SSF treatment.

Both cellulase cocktails, Cellic CTec2^®^ and Cellic CTec3^®^, were characterized for protein content and cellulase activity by the Bradford method [51] and the filter paper unit IUPAC method [52], respectively. The protein content and cellulase activity were 97.23 g/L and 126.5 FPU/mL for Cellic CTec2^®^, and 109.7 g/L and 175.65 FPU/mL for Cellic CTec3^®^. The full details regarding lignocellulosic hydrolysates, the growth media, and the fermentation conditions are described in [50]. The composition of the Norway spruce pretreated pulp and the derived lignocellulosic hydrolysates used in this study is provided in the Supplementary Material, Table S1.

### Bioreactor cultivations

The cultivation was performed in three steps: (1) the agar plate cultivation to prepare fresh spore suspension, (2) the pre-culture in nutrient rich media in Erlenmeyer shake flasks, and (3) the bioreactor cultivation in the nitrogen limited lipid production media.

The bioreactor cultivations comprised five different processes conducted in two independent biological replicates: (1) fermentation process with glucose (glucose control: bioreactors B01 and B02), (2) SSF without the addition of enzymes (SSF control: bioreactors B03 and B04), (3) SSF with the addition of enzyme (SSF: bioreactors B05 and B06), (4) separate hydrolysis and fermentation with Excello 90 lignocellulosic hydrolysate (SHF hydrolysate H1: bioreactors B07 and B08), and (5) separate hydrolysis and fermentation with NMBU BALI™ lignocellulosic hydrolysate (SHF hydrolysate H2: bioreactors B09 and B10). Sampling timepoints in the bioreactors were at: 2.5, 16, 24, 48, 72, 96, 120, 144, 168, 192 and 216 h.

All media had in common the following nutrients in g/L [40, 53]: yeast extract 3, KH_2_PO_4_ 7, Na_2_HPO_4_ 2, MgSO_4_·7H_2_O 1.5, CaCl_2_·2H_2_O 0.1, FeCl_3_·6H_2_O 0.008, ZnSO_4_·7H_2_O 0.001, CoSO_4_·7H_2_O 0.0001, CuSO_4_·5H_2_O 0.0001, and MnSO_4_·5H_2_O 0.0001. The carbon source varied between treatments. For all the liquid media (pure glucose and lignocellulosic hydrolysates) the glucose content was fixed to 80 g/L. For the SSF treatment, the solid loading of cellulose-rich BALI™ pulp was 38 g/L, equivalent to 37 g/L glucose. The enzyme loading of Cellic CTec3^®^ in SSF was 15.79% w/w (g of enzyme solution per g of pulp). High carbon-to-nitrogen ratio was used in order to induce the lipid accumulation in the oleaginous *Mucor circinelloides*.

### Estimation of cell dry weight

The biomass was separated from the culture supernatant by centrifugation and washed thoroughly with distilled water using the Millipore vacuum filtration system. SSF and SHF samples were washed three times by centrifugation. To determine the cell dry weight, 10 mL of culture broth was used for biomass washing, and the washed biomass was frozen and lyophilized until constant weight in a FreeZone 2.5 freeze-dryer (Labconco, USA) at − 50 °C and 0.01 mbar pressure. All samples were stored at − 20 °C until analysis.

For SSF processes (for bioreactors B03-B04: cellulose-rich pulp control, and B05-B06: SSF), the amount of fungal biomass was estimated by a method based on the determination of glucosamine (GlcN) content in fermented material. In the first step, alkali insoluble material (AIM) was prepared according to Zamani et al. [54]: 0.5 M NaOH solution (3 mL) was added to approx. 30–50 mg of each dried fermented sample and heated at 90 °C for 16 h. Subsequently, samples were centrifuged (5000 rpm, 10 min) and washed with distilled water (5 × 5 mL). Supernatants were removed, and obtained AIMs were dried at 70 °C for 48 h. Then, AIMs were mixed with 5 mL of 6 M hydrochloric acid and incubated at 100 °C for 12 h. Afterward, samples were processed according to the 3-methyl-2-benzothiazolinone-hydrazone hydrochloride (MBTH) colorimetric method described by Aidoo et al. [55], modified by Slaný et al. [56]. The amount of GlcN was assayed colorimetrically at 650 nm absorbance maximum and calculated subsequently according to the standard calibration curve. Analysis was performed in two independent technical replicates for each fermented sample.

### Preparation of cell suspension, supernatant, and fungal biomass for vibrational spectroscopy analyses

The biomass was separated from the supernatant (culture broth) by transferring the cell suspension with plastic Pasteur pipettes into 15 mL Falcon tubes, followed by centrifugation at 13,500 rpm for 15 min at 4 °C. The biomass from Falcon tubes was washed three times with cold distilled water and filtered under vacuum using Whatman No. I filter paper (GE Whatman, USA).

For FTIR microspectroscopy measurements, the washed biomass was resuspended and deposited on a zinc selenide (ZnSe) IR-transparent window.

For FTIR-ATR, the cell suspensions and the growth media reference solutions were measured in 15 mL Falcon tubes.

For FTIR-HTS measurements, approximately 2 mg of freeze-dried biomass was transferred into a 2 mL polypropylene tube containing 250 ± 30 mg of acid-washed glass beads and 0.5 mL of distilled water for further homogenization. The homogenization of fungal biomass was performed using a Precellys Evolution tissue homogenizer (Bertin Technologies, France) with the following setup: 5500 rpm, 6 × 20 s cycles. For FTIR-HTS measurements of dry biomass, 10 µL of homogenized fungal biomass was pipetted onto a silicon IR-transparent microplate. For FTIR-HTS measurements of supernatant, 10 µL of supernatant was pipetted onto a silicon IR-transparent microplate. For some samples, the supernatant was diluted up to 10 times.

For FT-Raman measurement of biomass, the freeze-dried biomass was deposited in glass vials. For FT-Raman measurement of cell suspension and supernatant, the cell suspension and supernatant were deposited in glass vials.

### FT-Raman spectroscopy analyses

The FT-Raman-HTS spectra were recorded in backscattering geometry using a MultiRAM FT-Raman spectrometer (Bruker Optik GmbH, Germany) equipped with a neodymium-doped yttrium aluminium garnet (Nd: YAG) laser (1064 nm, 9394 cm^− 1^) and a germanium detector cooled with liquid nitrogen. Samples were measured in glass vials using a High Throughput Screening (HTS) Mapping Stage, employing a 2.5 mm aperture and a collecting mirror objective. Spectra were recorded over the range of 3785–50 cm^− 1^, at 1000 mW laser power, using Blackman–Harris 4-term apodization. The OPUS 8.1 software (Bruker Optik GmbH, Germany) was used for data acquisition and instrument control.

For measurement of freeze-dried biomass, 5–10 mg of sample was deposited in a 400 µL flat-bottom glass insert vial for gas chromatography. The sample vials were placed in a 96-well microplate, the microplate was placed in a high throughput screening (HTS) stage, and the laser was focused on the bottom of the vial. The spectra were recorded with a total of 512 scans, spectral resolution of 8 cm^− 1^, with a digital resolution of 1.928 cm^− 1^. Each biomass sample, except the 2.5 h samples for bioreactors B01 & B02 (glucose), B07 & B08 (Excello 90 hydrolysate), and B09 & B10 (NMBU BALI hydrolysate), for which there was not enough biomass, was analysed in three technical replicates, resulting in 312 spectra.

For measurement of supernatant and cell suspension, 1 mL of sample was deposited in a 2 mL gas chromatography vial. The sample vials were placed in a 48-well microplate, the microplate was placed in a high throughput screening (HTS) stage, and the laser was focused on the bottom of the vial. The spectra were recorded with a total of 4096 scans, spectral resolution of 16 cm^− 1^, with a digital resolution of 3.857 cm^− 1^. Each supernatant and cell suspension sample was analysed in three technical replicates, resulting in 330 spectra each for the two (supernatant and cell suspension) spectral datasets.

### FTIR spectroscopy analyses

The FTIR-HTS transmittance spectra were measured using a High Throughput Screening eXTension (HTS-XT) unit coupled to a Vertex 70 FTIR spectrometer (both Bruker Optik, Germany), equipped with a globar mid-IR source and a deuterated triglycine sulfate (DTGS) detector. 10 µL of either supernatant or homogenized biomass was pipetted onto an IR-transparent 384-well silica microplate and dried at room temperature for two hours. The HTS-FTIR spectra were recorded with a total of 64 scans, spectral resolution of 6 cm^− 1^, digital spacing of 1.928 cm^− 1^, over the range of 4000–400 cm^− 1^, and an aperture of 5 mm. Spectra were recorded as the ratio of the sample spectrum to the spectrum of the empty IR-transparent microplate. Each sample was analysed in three technical replicates, resulting in 330 spectra each for the two (supernatant and biomass) spectral datasets. The OPUS software (Bruker Optik GmbH, Germany) was used for data acquisition and instrument control.

The FTIR microspectroscopy (FTIRµ) transmittance spectra were measured using a Hyperion 3000 IR microscope coupled to the Vertex 70 FTIR spectrometer (both Bruker Optik, Germany), equipped with a globar mid-IR source, a liquid nitrogen-cooled mercury cadmium telluride (MCT) 128 × 128 focal plane array detector system, and a computer-controlled x/y/z stage. Approximately 1–2 mg of washed biomass was resuspended in 0.5 mL of distilled water and deposited onto a 1 mm thick and 25 mm diameter IR-transparent ZnSe window, then dried at room temperature for two hours. The spectra were recorded with a total of 128 scans in the 3845–900 cm^− 1^ spectral range, with a spectral resolution of 8 cm^− 1^ and digital spacing of 3.8522 cm^− 1^. The samples were measured using a ×15 objective. Background spectra were recorded at the start of each measurement (one per sample) by measuring a sample-free area of the ZnSe microscope slide. Only samples from the last timepoint of fermentation (216 h) were measured. For each sample, four hyperspectral tiles (each consisting of a 128 × 128 pixel image) were recorded, resulting in 10 composite (4-tile) hyperspectral images. The OPUS software (Bruker Optik GmbH, Germany) was used for data acquisition and instrument control.

The FTIR-ATR spectra were measured using an IN350-T two-reflection diamond ATR fibre-optic probe coupled to a Matrix-MF FTIR spectrometer (both Bruker Optik, Germany), equipped with a globar mid-IR source and a liquid nitrogen-cooled MCT detector. The ATR probe was submerged into 15 mL Falcon tubes containing either a cell suspension or a growth media reference solution. The ATR-FTIR spectra were recorded with a total of 64 scans, spectral resolution of 4 cm^− 1^ and digital spacing of 1.928 cm^− 1^, over the range of 2500–850 cm^− 1^. Spectra were recorded as the ratio of the sample spectrum to the spectrum of the sample-free setting. Each sample was analysed in three technical replicates, resulting in 330 spectra. The OPUS software (Bruker Optik GmbH, Germany) was used for data acquisition and instrument control.

### Light microscopy

Micrographs were obtained from fresh biomass taken directly from the culture (no washing) on a glass microscope slide in brightfield mode with a DM6B microscope (Leica Microsystems, Wetzlar, Germany).

### Reference chemical analyses

To create the FTIR-ATR-based regression model for estimation of glucose, a set of reference solutions was prepared. The concentration of glucose was in the range from 0 to 80 g·L^− 1^, with a step of 10 g·L^− 1^, while the concentration of phosphate salts was 0, 0.5, 1.0, 2.0, and 3.0 standard concentrations (where 1.0 standard concentration was 7 g·L^− 1^ KH_2_PO_4_ and 2 g·L^− 1^ Na_2_HPO_4_) and the concentrations of ethanol were 0.05, 0.10, 0.25, 0.50, and 1.00%_v/v_. In addition, for the chemical characterisation of the cell suspension, a set of reference aqueous solutions of mannose and xylose (5 g·L^− 1^ and 60 g·L^− 1^) were measured by FTIR-ATR. The compounds were purchased from Merck-Sigma-Aldrich (Darmstadt, Germany) and used without further purification.

For the monitoring of glucose and ethanol concentrations, the supernatant was analysed on a Cedex^®^ Bio Analyzer (Roche) and used according to the producer [57]. The total lipid yields were determined as total fatty acid methyl esters (FAME) yields by conducting direct transesterification. Direct transesterification was performed according to Lewis et al. [58], with modifications according to Langseter et al. [41]. The lipid extraction and gas chromatography with flame ionization detector (GC-FID) analysis were performed for sampling points 72, 120, 168, and 216 h in two technical replicates, using a gas chromatography 7820 A System (Agilent Technologies, USA), equipped with an Agilent J&W 121–2323 DB-23 column, 20 m × 180 μm × 0.20 μm, and a flame ionization detector (FID). The detailed description was reported previously [50].

### Spectral preprocessing and data analysis

All preprocessing methods, data analyses, and statistical analyses were performed using Aspen Unscrambler 14.2 (Aspen Technology, Inc, Bedford MA, USA) and Orange data mining toolbox version 3.26 (University of Ljubljana, Slovenia) [59, 60].

The FTIR-ATR spectral dataset was preprocessed by truncation of data to 1300–850 cm^− 1^ spectral region, baseline correction (baseline offset), and peak normalisation (at 850 cm^− 1^). FTIR-HTS spectral datasets were preprocessed by extended multiplicative signal correction (EMSC), an MSC model extended by linear, quadratic and cubic components [61, 62]. FT-Raman-HTS spectral datasets were converted into second derivatives by using the Savitzky–Golay algorithm (polynomial 2, window size 19, derivative order 2), truncation of data to 3200 − 2600 and 1800–600 cm^− 1^ regions, and MSC preprocessing.

Significance testing was conducted with ANOVA and Student’s t-test. The levels of significance were set at *p* < 0.05, *p* < 0.01, and *p* < 0.001, with results exhibiting *p* < 0.001 being considered highly statistically significant.

Biochemical similarities between samples were estimated by using principal component analysis (PCA). PCA was conducted on FTIR-HTS data of biomass and FT-Raman-HTS data of biomass, supernatant, and cell suspension.

Partial least squares regression (PLSR) was used to establish several regression models. The PLSR model for glucose estimation by FTIR-ATR was established by using a set of reference glucose concentrations as a Y matrix (response), which was regressed onto an X matrix containing FTIR-ATR spectra of reference glucose solutions (predictors). The optimal number of PLSR components (i.e., PLSR factors) of the calibration models (*A*_*Opt*_), root-mean-square error (RMSE), and coefficient of determination (*R*^*2*^) were calculated, and the optimal model was selected based on the lowest *A*_*Opt*_ having an insignificantly higher RMSE than the model with the minimum RMSE. The training set consisted of data corresponding to glucose concentrations of 0, 20, 30, 50, 60, and 80 g·L^− 1^, while the validation set consisted of data corresponding to glucose concentrations of 10, 40, and 70 g·L^− 1^. The established PLSR model was used to estimate glucose concentration in supernatant samples measured by FTIR-ATR.

The PLSR models for glucose and lipid content estimation by FT-Raman and FTIR-HTS were established by using a data set of reference glucose concentrations (via Cedex^®^ Bio Analyzer) or lipid content (via GC-FID) as a Y matrix (response), which was regressed onto an X matrix containing FT-Raman or FTIR-HTS spectra of cell suspension, biomass or supernatant (predictors). The optimal number of PLSR components (i.e., PLSR factors) of the calibration models (*A*_*Opt*_), root-mean-square error (RMSE), and coefficient of determination (*R*^*2*^) were calculated, and the optimal model was selected based on the lowest *A*_*Opt*_ having an insignificantly higher RMSE than the model with the minimum RMSE. The independent test validation was performed separately for each of the mentioned datasets by dividing the training and validation sets so that each set contained data from one of the two independent biological replicates. The established PLSR models were used to estimate glucose concentration in supernatant samples measured by FTIR-HTS, as well as in supernatant or cell suspension samples measured by FT-Raman. Moreover, the established PLSR models were used to estimate lipid content in biomass or cell suspension samples measured by FT-Raman, as well as in biomass samples measured by FTIR-HTS.

## Results and discussion

### Biomass and lipid production

**Fig. 1:**
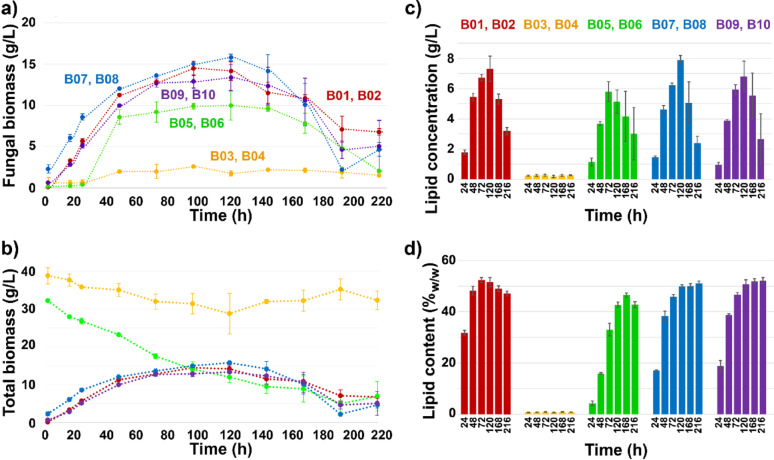
**a** Fungal biomass concentration (g/L), **b** total biomass concentration (g/L), **c** lipid concentration (g/L), and **d** lipid content (as percentage of dry biomass, %_w/w_) for bioreactors B01-B02: glucose control; B03-B04: cellulose-rich pulp control; B05-B06: SSF; B07-B08: SHF hydrolysate H1; B09-B10: SHF hydrolysate H2

The fermentation experiments were conducted with five different growth media in duplicates: glucose control media (B01 & B02), cellulose-rich BALI™ pulp control media (B03 & B04), SSF media (B05 & B06), and two types of hydrolysate (SHF) media (B07 & B08 and B09 & B10), both originally obtained from the cellulose-rich BALI™ pulp as in the SSF. The utilization of growth media by *Mucor circinelloides* was observed in all cases, except for the bioreactors B03 and B04 with cellulose-rich pulp control media (Fig. 1a). In this treatment, fungal growth was likely promoted solely by the yeast extract of the medium and leftover nutrients from the preculture inoculum. Then, it remained minimal, while the total solids (mainly pulp) remained unchanged (Fig. 1b). This was expected since *Mucor circinelloides* lacks a complete set of enzymes for an efficient breakdown of cellulose [63]. Briefly, for the other treatments, fungal growth was exponential during the first 48 h, followed by the lipid accumulation stationary phase. The maximum production of 15.83 g/L of fungal biomass was obtained for SHF hydrolysate H1 (Excello 90). The control glucose-based medium provided 14.50 g/L of *Mucor circinelloides* biomass. Biomass production in the SSF strategy reached 9.96 g/L at 120 h. However, it should be noted that the starting carbon concentration in the SSF media was much lower than in the other media. Based on the maxima of biomass concentrations, it is apparent that the most suitable cultivation time is 120 h, since a rapid decrease in biomass concentration was observed after this timepoint. More details about the SHF processes were reported previously [50]. *M. circinelloides* is a dimorphic fungus that can grow in filamentous form or yeast-like cells depending on the conditions. In this study, the morphology in all treatments was the filamentous form, as can be seen in microscopy images (Supplementary Material, Figure S2).

Statistical analysis indicated that there were no significant differences (*p* > 0.05) between the two independent biological replicates for any of the processes, for both total lipid content and total biomass (Supplementary Material, Table S2). This demonstrates a high degree of reproducibility of the processes. With respect to differences between the processes, there was a highly significant statistical difference (*p* < 0.001) observed for both total lipid content and total biomass. However, no significant differences were observed between the glucose control medium and the two SHF media, demonstrating the high quality of the SHF media for supporting fungal fermentation.

For all fermentations, total lipids (as fatty acid methyl esters) and fatty acid profiles were determined by direct transesterification and GC-FID analysis, as reported previously [50]. Compared to the glucose control media, fermentation in the hydrolysate media resulted in similar lipid production, while SSF resulted in approx. 20% lower production (Figs. 1c). Nevertheless, lipid accumulation over fungal dry weight was satisfactory, with approx. 50%_w/w_ for all SHF and SSF treatments (Fig. 1d).

In all cases, the maximum lipid production was obtained at approx. 72–120 h. At those timepoints for the glucose and hydrolysate treatments, the maximum lipid production yields ranged from 0.091 to 0.153 g/g. This yield could not be calculated for the SSF and cellulose-rich pulp control treatments due to the inability to track and quantify the total glucose released from the cellulose-rich material. After 120 h, the lipid yields declined sharply since the fungal biomass continued consuming glucose for maintenance without producing additional biomass or lipids, as described in our previous study [50]. Papanikolaou et al. estimated a maximum lipid yield from glucose of 0.32 g/g based on stoichiometric calculations [64]. However, lipid yields through glucose fermentation with oleaginous yeasts are commonly 0.22–0.27 g/g, while slightly lower in filamentous fungi with values around 0.03–0.19 g/g, including Mucoromycota species [2, 65]. Note that these values depend not only on the microorganisms’ conversion efficiency but also on the maximum accumulation capacity and carbon allocation to other products of each species. Therefore, our results are above the average expected for filamentous fungi of the Mucoromycota. As expected, biomass growth was insignificant in lignocellulose control media since *Mucor circinelloides* lacks a complete set of enzymes for an efficient breakdown of cellulose [63].

Regarding the fatty acid profile, the SSF and glucose fermentations had similar fatty acid profiles. In contrast, the lipids from the hydrolysate media were slightly different compared to those from the glucose control media, with hydrolysate-media lipids containing more monounsaturated fatty acids (in particular oleic acid) and fewer polyunsaturated fatty acids. More details are available in [50].

### FT-Raman spectroscopy of cell suspension

**Fig. 2:**
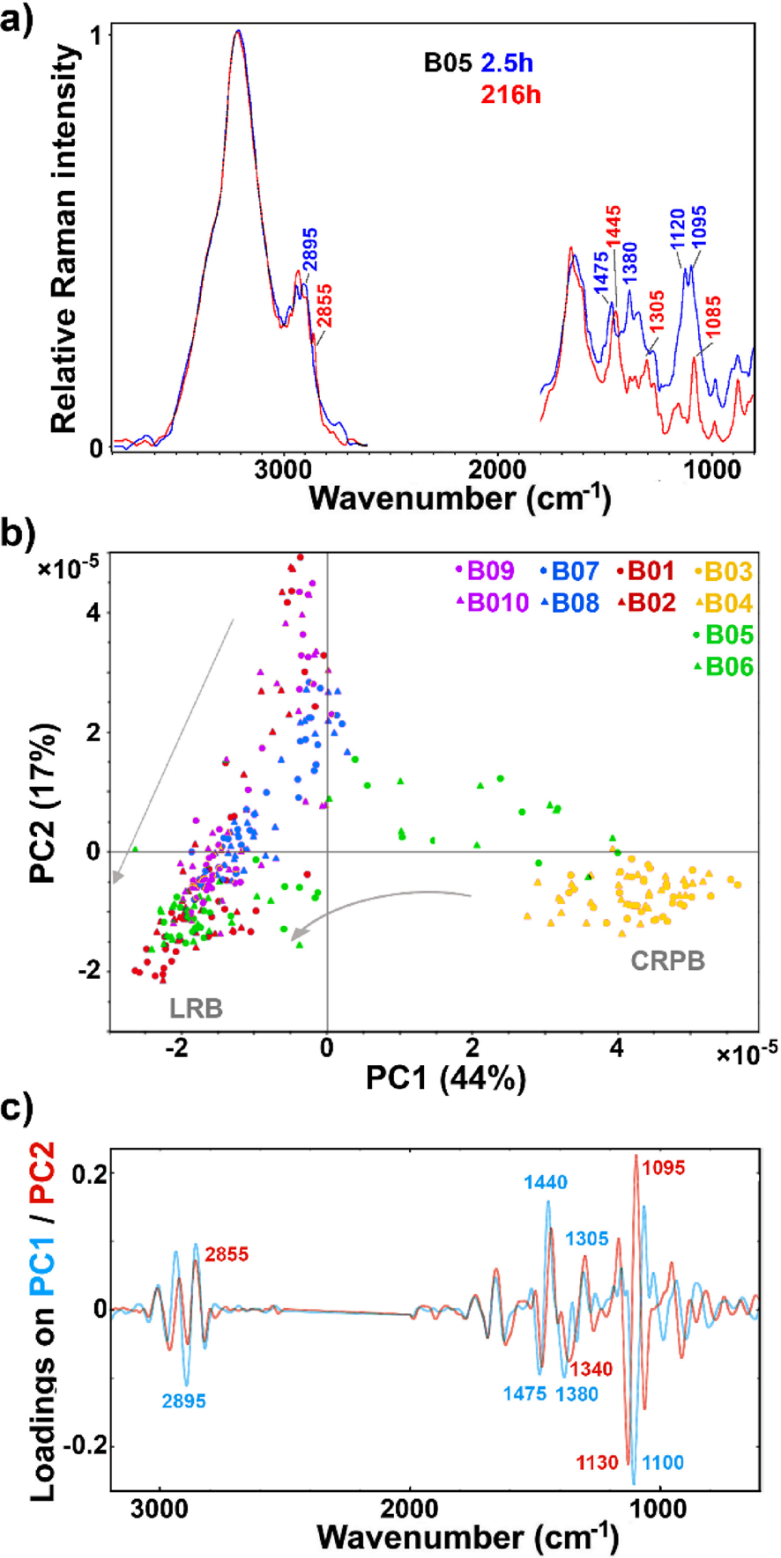
FT-Raman-HTS spectra of cell suspension: **a** spectra of SSF (B05) at 2.5 and 216 h. **b** PCA score plots of PC1 and PC2, and **c** the first two loading vectors. Score plots are labelled according to bioreactors: B01-B02: glucose control; B03-B04: cellulose-rich pulp control; B05-B06: SSF; B07-B08: SHF hydrolysate H1; B09-B10: SHF hydrolysate H2. Arrows indicate time progression, from start of the bioprocesses to the endpoints. CRPB: cellulose-rich pulp biomass, LRB: lipid-rich biomass. The explained variances for the first five principal components are 43.8%, 16.8%, 6.9%, 4.3%, and 1.7%

**Fig. 3:**
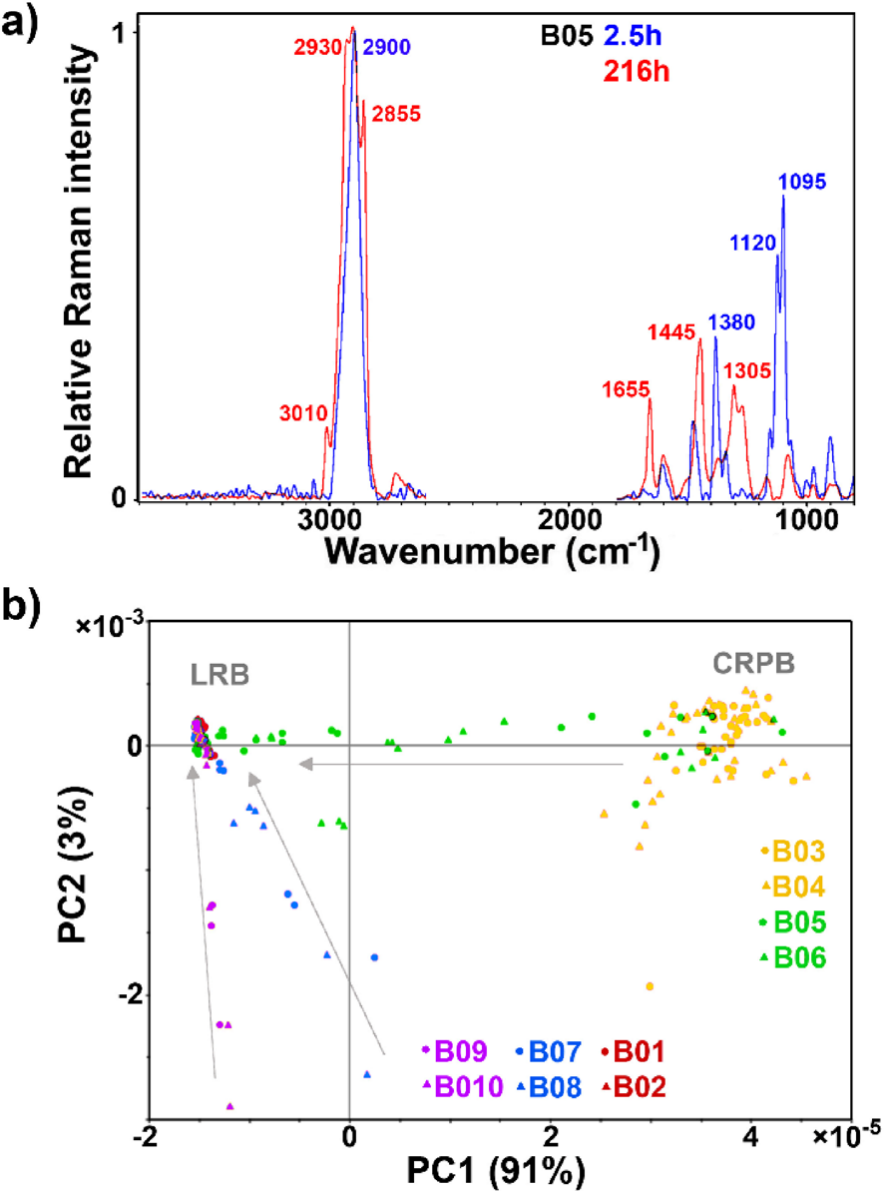
FT-Raman-HTS spectra of biomass: **a** spectra of SSF (B05) at 2.5 and 216 h. **b** PCA score plots of PC1 and PC2. Score plots are labelled according to bioreactors: B01-B02: glucose control; B03-B04: cellulose-rich pulp control; B05-B06: SSF; B07-B08: SHF hydrolysate H1; B09-B10: SHF hydrolysate H2. Arrows indicate time progression, from the start of the bioprocesses to the endpoints. CRPB: cellulose-rich pulp biomass, LRB: lipid-rich biomass. The explained variances for the first five principal components are 90.8%, 3.3%, 0.9%, 0.5%, and 0.3%

FT-Raman spectra of cell suspensions show strong signals related to biomass, either the cellulose-rich pulp substrate or the fungal biomass (Fig. 2a). Spectra are very similar to those of dry biomass (Fig. 3a), with additional signals of water at 3200 and 1640 cm^− 1^ (OH stretching and HOH bending vibrations, respectively). The PCA score plot (Fig. 2b) shows that the SSF process can be monitored by FT-Raman since these water bands are fairly well separated from the main bands related to the cellulose-rich pulp substrate (at 2895, 1475, 1380, 1120, and 1095 cm^− 1^) and the fungal biomass (at 2855, 1445, and 1305 cm^− 1^).

The measurements of cell suspensions demonstrate the principal difference between FTIR and FT-Raman spectroscopies as PAT tools for monitoring and control of SSF processes. Water has strong absorbance bands in the mid-infrared spectral range, which hampers FTIR measurements of cell suspensions. This problem can be tackled by removal of water and conducting transmittance measurements of dry samples, as in FTIR-HTS. However, this approach limits FTIR to an at-line monitoring tool, with a time delay required for preparation of dry films for FTIR-HTS. Another approach is reflectance measurements of liquid samples with a very short effective path length of the IR radiation, as in FTIR-ATR. Although this approach allows the use of FTIR as an on-line monitoring tool via fibre-optic submerged probes, it also has a significant limitation. Namely, the short effective path length of approx. 2–10 μm enables only measurement of liquid growth media, and not of suspended biomass. While FTIR-ATR and FTIR-HTS can be very effective tools for monitoring growth media, particularly in the production of extracellular metabolites [24], they have very limited value in monitoring of the SSF process. As demonstrated here, soluble saccharides during the SSF process are present at low concentrations in the growth media, since monosaccharides are gradually produced by hydrolysis and almost immediately utilised by fungi. In the case of intracellular SCO production by SSF, monitoring growth media provides less valuable information than direct measurement of biomass. FT-Raman spectroscopy enables direct measurement of both cellulose-rich pulp substrate and fungal biomass in cell suspension. Moreover, FT-Raman is able to measure signals specific to SCO production, namely lipid-related signals at 2855 and 1305 cm^− 1^. An important advantage of Raman spectroscopy over FTIR is the ability to measure through glass, as we have demonstrated recently by non-invasive real-time monitoring of biomass production, production of intracellular SCO and carotenoids, and carbon substrate utilisation in the fermentation media during fermentation of oleaginous and carotenogenic microorganisms [27].

**Table 1:** PLSR coefficients of determination (R^2^), root mean square errors (RMSE) and number of components (*A*_*opt*_) for determination of glucose concentration (g/L) and lipid content (as percentage of dry biomass, %_w/w_), based on FT-Raman spectra of cell suspension

FT-Raman cell suspension	A_opt_	*R* ^2^	RMSE
Glucose (g/L)	2	0.78	14.28 (15.4%)
Lipid content (%_w/w_)	4	0.87	7.42 (14.4%)

Quantitative estimates of glucose in the cultivation media and lipid content in the biomass were obtained by PLSR analyses of the FT-Raman measurements from the cell suspension (Table 1). The results show high level of correlation between the FT-Raman spectra and the reference measurements. The RMSE value for glucose assessment in this study was substantially higher (RMSE = 15%), compared to previous results obtained for yeast fermentation (RMSE = 3%) [27]. However, it should be noted that the current RMSE was calculated using an independent test set rather than cross-validation, and the PLSR model was developed to cover both SSF and SHF fermentation types, rather than a single fermentation process. Since the model was developed using a relatively small dataset, we believe its performance can be further improved by incorporating additional data. Furthermore, the accuracy could be improved by conducting measurements under real-time dynamic conditions (such as using a flow cell coupled to a bioreactor, optical window on a bioreactor, or a submerged-probe measurement), rather than under stationary conditions, where samples are collected in vials and biomass settles at the bottom. In the current setup, Raman measurements are performed by focusing the laser light on the bottom of the vial, which may contribute to the relatively high RMSE observed for glucose assessment due to the presence of sedimented biomass. The results for lipid content were more than satisfactory (RMSE = 14%), especially considering the complexity of the processes and the aforementioned limitations of the model. Further refinement and improvement of the model are expected to lead to even better performance. The low number of components (PLS factors) required to build the glucose and lipid content models indicates that the developed models are highly stable and reliable. The model for lipid concentration (g/L) showed relatively poor performance (R^2^ = 0.63), likely due to the fact that measurements were made in vials where biomass sediments. Improved results are expected when using fully mixed cell suspensions, where the concentration of biomass can be assessed more accurately.

In this study, the configuration used was specifically optimized for high-throughput measurements aimed at near-real-time at-line monitoring. Notably, the same instrument, with a slightly different configuration, can also be applied for online monitoring, as demonstrated in the previous study [27]. In fact, in the online monitoring configuration, spectral acquisition can be achieved even faster due to improved signal-to-noise ratio, which results from the shorter and less complex optical path of the backscattered light. In this configuration, the backscattered light travels a more direct route to the detector, minimizing IR losses and distortions during the propagation. This study thus highlights FT-Raman spectroscopy as an ideal PAT tool for both online and at-line monitoring of SSF processes for intracellular SCO production.

### FT-Raman spectroscopy of biomass and supernatant

FT-Raman-HTS spectra show huge differences between fungal biomass and cellulose-rich pulp substrate (Fig. 3a). The cellulose-rich pulp substrate is characterised by signals at 2900 cm^−1^ (C–H stretching), 1470 cm^−1^ (CH_2_, COH vibrations), 1380 and 1340 cm^−1^ (CH_2_ vibrations), and 1120 and 1095 cm^−1^ (C–O–C glycosidic stretching) [66]. Lignin-specific aryl ring vibrations, at approx. 1605 and 1510 cm^−1^ [67], are relatively broad and weak due to the low amount of lignin in the cellulose-rich pulp substrate (approx. 3%) [68, 69]. Lipid-rich fungal biomass is characterised by signals at 3010–2850 cm^− 1^ (=C–H and –C–H stretching vibrations), at 1655 cm^− 1^ (C=C stretching), and at 1460–1440 and 1380–1320 cm^− 1^ (C–C, C–O, C–O–C, C–N, CH, COH vibrations) [10]. In general, monitoring of SSF and SHF processes is relatively simple due to extensive spectral differences between the cellulose-rich pulp substrate and the lipid-rich fungal biomass.

The PCA score plot (Fig. 3b) shows that the two hydrolysates differ regarding biomass at the early growth stage. The substantial differences observed between the spectra of glucose biomass and hydrolysate biomasses at the initial timepoints are primarily attributed to fluorescence, and not the actual chemical differences. Fluorescence distorts the baseline in FT-Raman spectra by introducing broad, intense background signals that obscure the true Raman scattering peaks, making it challenging to obtain chemical information from the spectral data. This fluorescence is likely caused by lignin particles (originating from the hydrolysates) that are being adsorbed onto the fungal biomass. The amount of these lignin particles can be considered minor, as will be discussed later in connection with the FTIR-HTS spectroscopy results. Regarding the end timepoints, a similar type of lipid-rich fungal biomass was obtained from the four processes: SSF, hydrolysates H1 and H2, and glucose control. These results demonstrate yet again the considerable complementarity of FTIR and FT-Raman spectroscopy data.

**Fig. 4:**
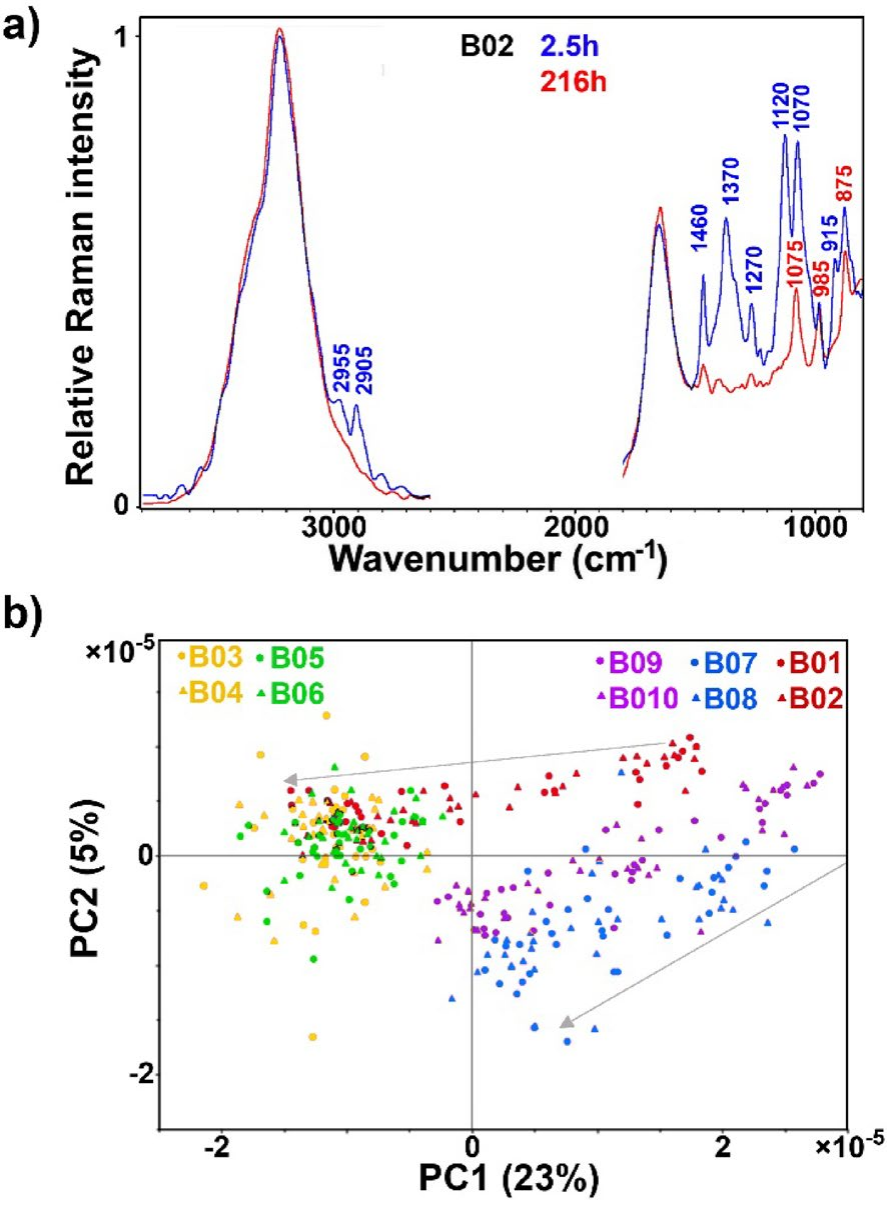
FT-Raman-HTS spectra of supernatant: **a** spectra of glucose control process (B02) at 2.5 and 216 h. **b** PCA score plots of PC1 and PC2. Score plots are labelled according to bioreactors: B01-B02: glucose control; B03-B04: cellulose-rich pulp control; B05-B06: SSF; B07-B08: SHF hydrolysate H1; B09-B10: SHF hydrolysate H2. Arrows indicate time progression, from start of the bioprocesses to the endpoints. The explained variances for the first five principal components are 23.0%, 5.2%, 4.3%, 3.9%, and 2.9%

FT-Raman-HTS spectra of supernatant can be used for monitoring glucose, but only at high concentrations present in SHF hydrolysates H1 and H2, and glucose control processes (Fig. 4a). At the start of these processes, the spectra are dominated by glucose-related signals at 2955, 2905, 1370, 1270, 1120, 1070, and 915 cm^− 1^, while at the end of the processes the dominant signals are phosphate-related at 1075 cm^− 1^ (H_2_PO_4_^−^), 985 cm^− 1^ (HPO_4_^2−^) and 875 cm^− 1^ (H_2_PO_4_^−^).

**Fig. 5:**
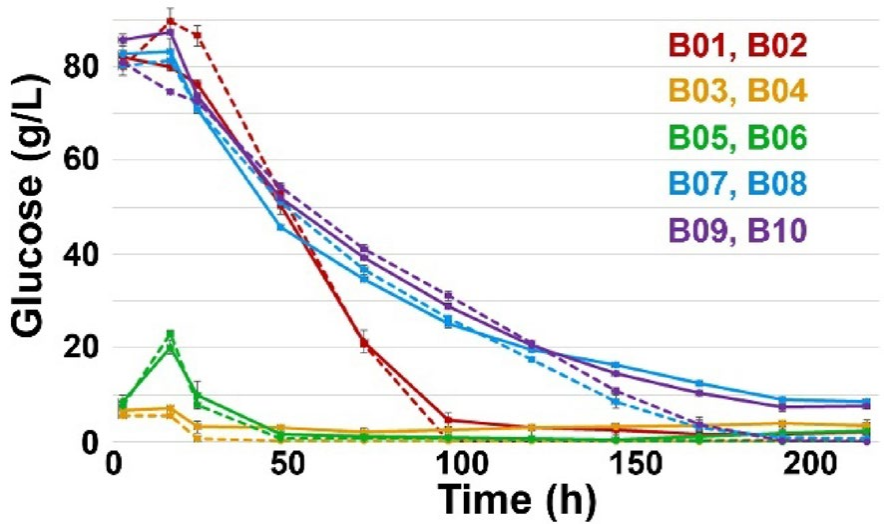
Glucose concentration at sampling points for bioreactors B01-B10, estimated by the FTIR-ATR PLSR model (solid line) and CEDEX reference measurement (dash line). B01-B02: glucose control; B03-B04: cellulose-rich pulp control; B05-B06: SSF; B07-B08: SHF hydrolysate H1; B09-B10: SHF hydrolysate H2

The PCA score plot (Fig. 4b) shows that supernatants of the hydrolysates and glucose control at the end of the processes are different. This is further confirmed by the subsequent FTIR-ATR and Cedex results (Fig. 5). The main difference is due to residual carbohydrates (mannose and xylose) present in the hydrolysate processes.

Quantitative estimates of glucose in the cultivation media and lipid content in the biomass were obtained by PLSR analysis of FT-Raman measurements from the supernatant and biomass, respectively (Table 2). The RMSE value for glucose assessment was substantially lower (RMSE = 11%) for supernatant measurement, compared to cell suspension measurement (Table 1, RMSE = 15%). This difference is likely due to the aforementioned presence of sedimented biomass in the vial during cell suspension measurements, a factor that is expected to be minimized under real-time dynamic conditions where the cell suspension is thoroughly mixed. The RMSE for lipid content was relatively high (RMSE = 16%), but this result is still satisfactory given that the model is very simple, utilizing only one component, and was developed with a relatively small dataset. Based on our previous studies on FT-Raman assessment of filamentous fungal biomass [10], we are confident that its performance can be further improved, with the potential to reach an RMSE in the 5–10% range.

**Table 2:** PLSR coefficients of determination (R^2^), root mean square errors (RMSE) and number of components (*A*_*opt*_) for determination of glucose concentration (g/L) based on FT-Raman spectra of supernatant, and lipid content (as percentage of dry biomass, %w/w) based either FT-Raman of FTIR-HTS spectra of biomass

	A_opt_	*R* ^2^	RMSE
Glucose (g/L) FT-Raman supernatant	2	0.90	9.83 (10.6%)
Lipid content (%_w/w_) FT-Raman biomass	1	0.84	8.43 (16.3%)
Lipid content (%_w/w_) FTIR-HTS biomass	1	0.90	6.76 (13.1%)

### FTIR-ATR spectroscopy of cell suspension

As expected, FTIR-ATR is well-suited for monitoring glucose in growth media, as we have demonstrated previously [11, 24]. For the determination of glucose in the growth media via FTIR-ATR, a PLSR model was built (Supplementary Material, Figure S3), trained and validated on measurements of reference glucose solutions. The PLSR model was very robust, with only two components, and with 0.99 R^2^ and 3.19 (3.99%) RMSE (both R^2^ and RMSE are for the independent validation). The results show high consistency between glucose estimates by the PLSR model and the reference Cedex method (Fig. 5). Even the relatively low glucose concentrations present during the early stages of the SSF process were accurately estimated by the FTIR-ATR-based PLSR model. It can be stated that the FTIR-ATR approach works well in the concentration range of 5–80 g/L. However, glucose concentrations during the late stage of the SSF process (48–216 h) are close to zero, and thus it cannot be expected that the FTIR-ATR approach will be useful for accurate monitoring at these low concentrations.

**Fig. 6:**
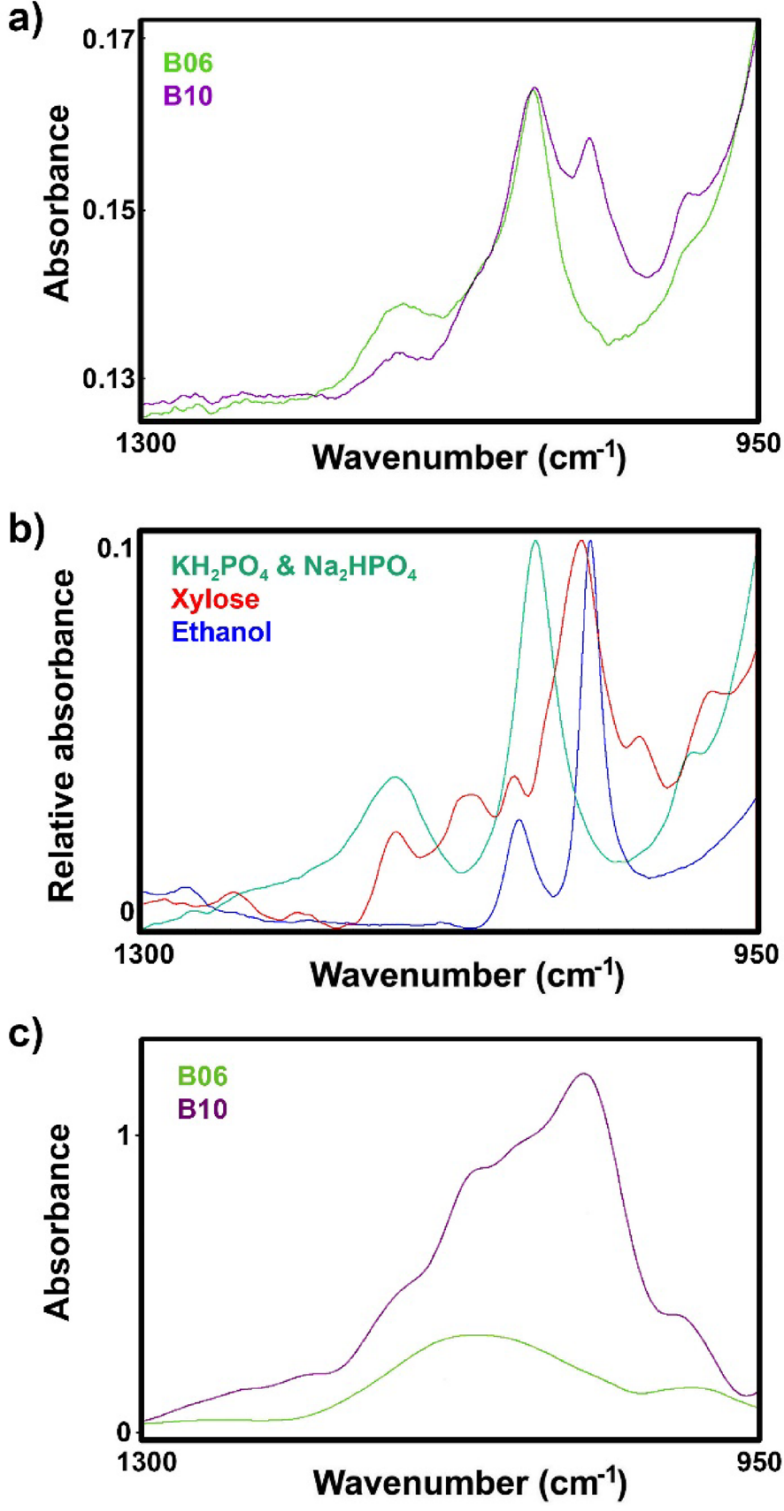
FTIR-ATR spectra of (**a**) cell suspensions for SSF (B06) and SHF hydrolysate H2 (B10) at 216 h, and (**b**) reference solutions of phosphate salts (KH_2_PO_4_ and Na_2_HPO_4_), xylose and ethanol (the spectra were normalised for better viewing). **c** FTIR-HTS spectra of supernatant for SSF (B06) and SHF hydrolysate H2 (B10) at 216 h

The FTIR-ATR spectra of hydrolysate media indicate that, although in the late stage of the process hydrolysate media are depleted of glucose, they still contain residual carbohydrates (Fig. 6). The residual carbohydrates are not present in the SSF media, and only the presence of phosphate salts can be observed (Fig. 6). The spectral range in Fig. 6 was limited to 1300–950 cm^− 1^ because dissolved analytes exhibit strong signals in this region, while at higher and lower wavenumbers, the spectra are dominated by the absorbance bands of water, specifically the H-O-H bending vibration around 1650 cm^− 1^ and the libration mode near 800 cm^− 1^. The difference between FTIR-ATR spectra at 216 h for SSF (B06) and hydrolysate H2 (B10) is consistent with the presence of residual xylose and produced ethanol in the hydrolysate [50]. Xylose and mannose are minor components in the starting hydrolysate (approx. 10% of total carbohydrates) [68, 69]. Interestingly, the results indicate that xylose is being utilised as a carbon source during SSF, while it is disregarded during SHF in favour of glucose. Previous studies have shown that *Mucor circinelloides* favours glucose assimilation over other carbon sources, though at lower glucose concentrations other carbohydrates, such as mannose and xylose, can be co-utilized as carbon sources [50, 70].

Other carbohydrates were not present in the training set of spectra for the PLSR model, and thus it is unsurprising that the model deviates from the correct results during the late-stage fermentation (144–216 h) of hydrolysate media (B07 & B08 and B09 & B10). Since this study focused specifically on the monitoring the SSF process and not the SHF process, the expansion of PLSR models to include SHF-related carbohydrates was considered outside the scope of this research. Thus, future FTIR-ATR-based PLSR models for monitoring of carbohydrates in lignocellulose hydrolysates for SHF should be trained on mannose and xylose reference solutions.

### FTIR-HTS spectroscopy of biomass and supernatant

**Fig. 7:**
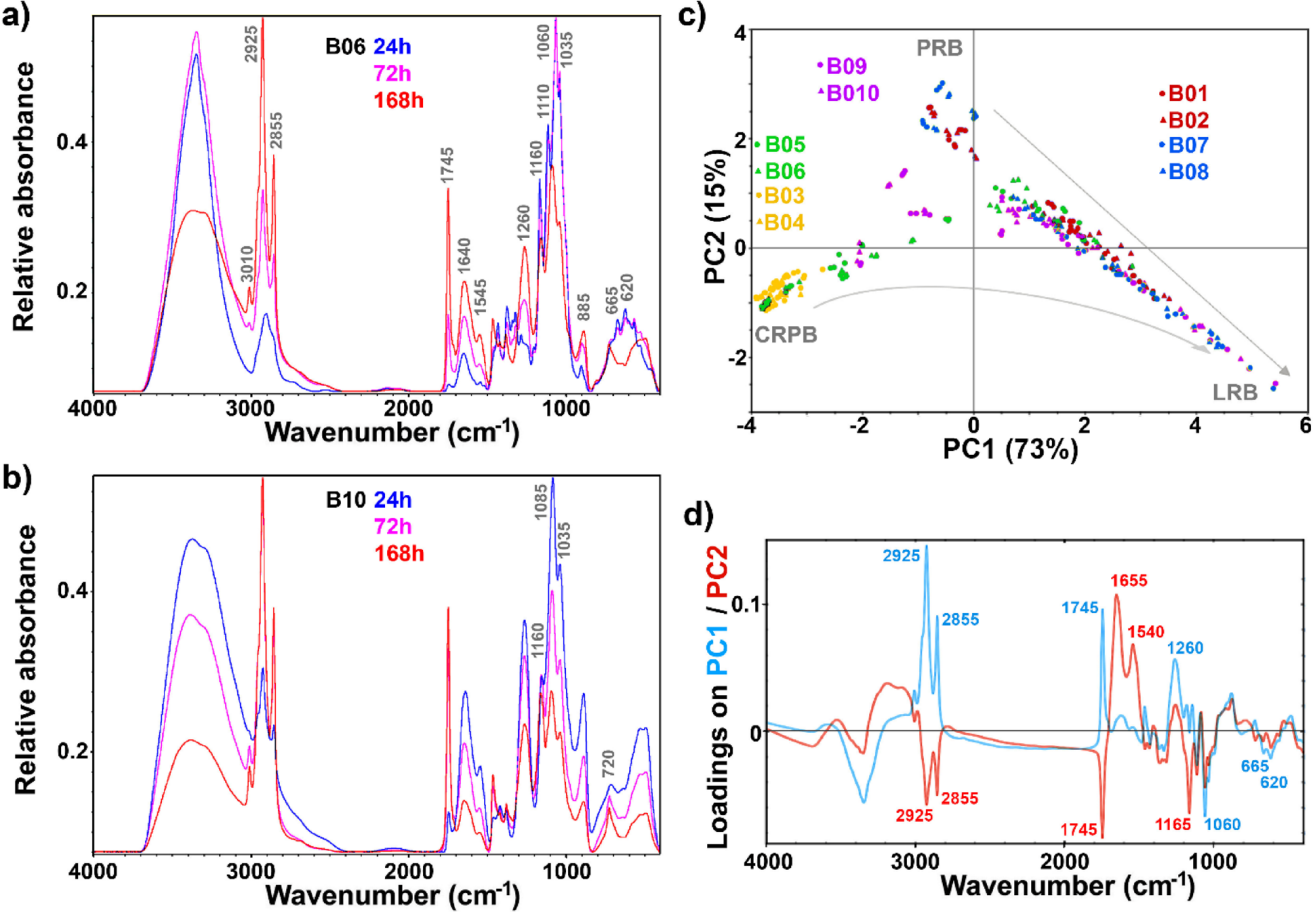
FTIR-HTS spectra of biomass: **a** spectra of SSF (B06) and **b** SHF hydrolysate H2 (B10) at 24, 72 and 168 h. **c** PCA score plots of PC1 and PC2, and **d** the first two loading vectors. Score plots are labelled according to bioreactors: B01-B02: glucose control; B03-B04: cellulose-rich pulp control; B05-B06: SSF; B07-B08: SHF hydrolysate H1; B09-B10: SHF hydrolysate H2. Arrows indicate time progression, from the start of bioprocesses to the endpoints. CRPB: cellulose-rich pulp biomass, PRB: protein-rich biomass, LRB: lipid-rich biomass. The explained variances for the first five principal components are 73.2%, 15.4%, 5.7%, 2.6%, and 1.0%

As mentioned for FTIR microspectroscopy, there are large differences between infrared spectra of fungal biomass and lignocellulose substrate. These specific differences can be exploited for monitoring the SSF process with FTIR-HTS spectroscopy, an extremely convenient at-line method. For example, FTIR-HTS spectra of biomass grown in hydrolysate H2 (Fig. 7b), obtained after 24, 72, and 168 h of cultivation, show progression from the protein-rich biomass, characterised by strong amide I (1640 cm^−1^) and amide II (1545 cm^−1^) bands, to the lipid-rich biomass, characterised by strong lipid-related bands at 3010–2850 cm^−1^ (=C–H and –C–H stretching vibrations), 1745 cm^−1^ (–C=O stretching in esters), and 1160 cm^− 1^ (C–O–C stretching in esters) [2, 11, 13]. In addition, the biomass has characteristic signals related to the biopolymers in the fungal cell wall: polyphosphates, characterised by signals at 1260 cm^−1^ (P=O stretching) and at 885 cm^−1^ (P-O-P stretching), as well as chitin, chitosan, and glucans, characterised by signals at 1085 and 1035 cm^−1^ (C–O and C–O–C stretching vibrations) [2, 11, 13]. Conversely, FTIR-HTS spectra of SSF biomass obtained from the corresponding timepoints (Fig. 7a) show that the dominant signals during early-stage cultivation are related to the lignocellulose substrate, with characteristic cellulose- and hemicellulose-related signals at 1425, 1375, and 1320 cm^−1^ (CH, CH_2_, CH_3_ vibrations) and at 1110, 1060, and 1035 cm^−1^ (C–O and C–O–C stretching vibrations) [71]. Even after 72 h, when the fungal biomass is clearly rich in lipids, the total biomass in the bioreactor is still predominantly lignocellulose. Only after 168 h are the FTIR spectra of the biomass largely devoid of cellulose signals, and they start to resemble the lipid-rich spectra of the biomass obtained from fermentation in hydrolysate H2.

The PCA score plot clearly shows the spectral differences between lignocellulose biomass, protein-rich fungal biomass, and lipid-rich fungal biomass (Fig. 7c), similar to the FT-Raman-HTS results (Fig. 3b). The two hydrolysates differ regarding fungal biomass at the early sampling points, while the biomass of the hydrolysate H2 process is more similar to the early glucose control biomass. The amount of lignin particles detected by FT-Raman spectroscopy (Fig. 3b) can be considered minor, since there are no substantial differences between the FTIR spectra of glucose biomass and hydrolysate biomasses at the initial timepoints. The end result of the four processes (SSF, hydrolysates H1 and H2, and glucose control) is a similar type of lipid-rich biomass. In general, these results corroborate our previous studies [2, 11, 13, 24, 41, 42], demonstrating once again that FTIR-HTS spectroscopy is a very rapid and economical method for the assessment of microbial biomass.

Quantitative estimates of lipid content in the biomass was obtained by PLSR analysis of FTIR measurements from the biomass (Table 2). Like the findings from FT-Raman measurements of the biomass, the RMSE for lipid content was relatively high (RMSE = 13%). However, this result remains satisfactory considering the simplicity of the model (only one component), and the fact that it was developed with a relatively small dataset. As with the FT-Raman model for biomass, our previous studies on FTIR assessment of filamentous fungal biomass [10] indicate that the model’s performance can be further improved, with the potential to achieve an RMSE in the 5–10% range.

Although we have shown previously that FTIR-HTS analysis of supernatant can provide similar results to FTIR-ATR [24], this was not the case in this study due to the low quality of the recorded spectra. Obtaining a thin plane-parallel film of solid material on an HTS microplate is a prerequisite for acquiring high-quality FTIR-HTS spectra. Unfortunately, for a number of samples, drying of supernatant on an HTS microplate resulted in glassy irregular clumps, due to the high crystallinity of phosphates and carbohydrates present in the supernatant. The end result was low-quality spectra, with scattering artefacts and large absorbance and baseline variations due to sub-optimal film formation [72]. Therefore, quantitative analysis of supernatant FTIR-HTS spectra was not performed. The FTIR-HTS spectra of supernatant at the endpoints of the processes support FTIR-ATR results regarding the presence of residual carbohydrates in the SHF media (Fig. 6c).

### FTIR microspectroscopy of biomass

**Fig. 8:**
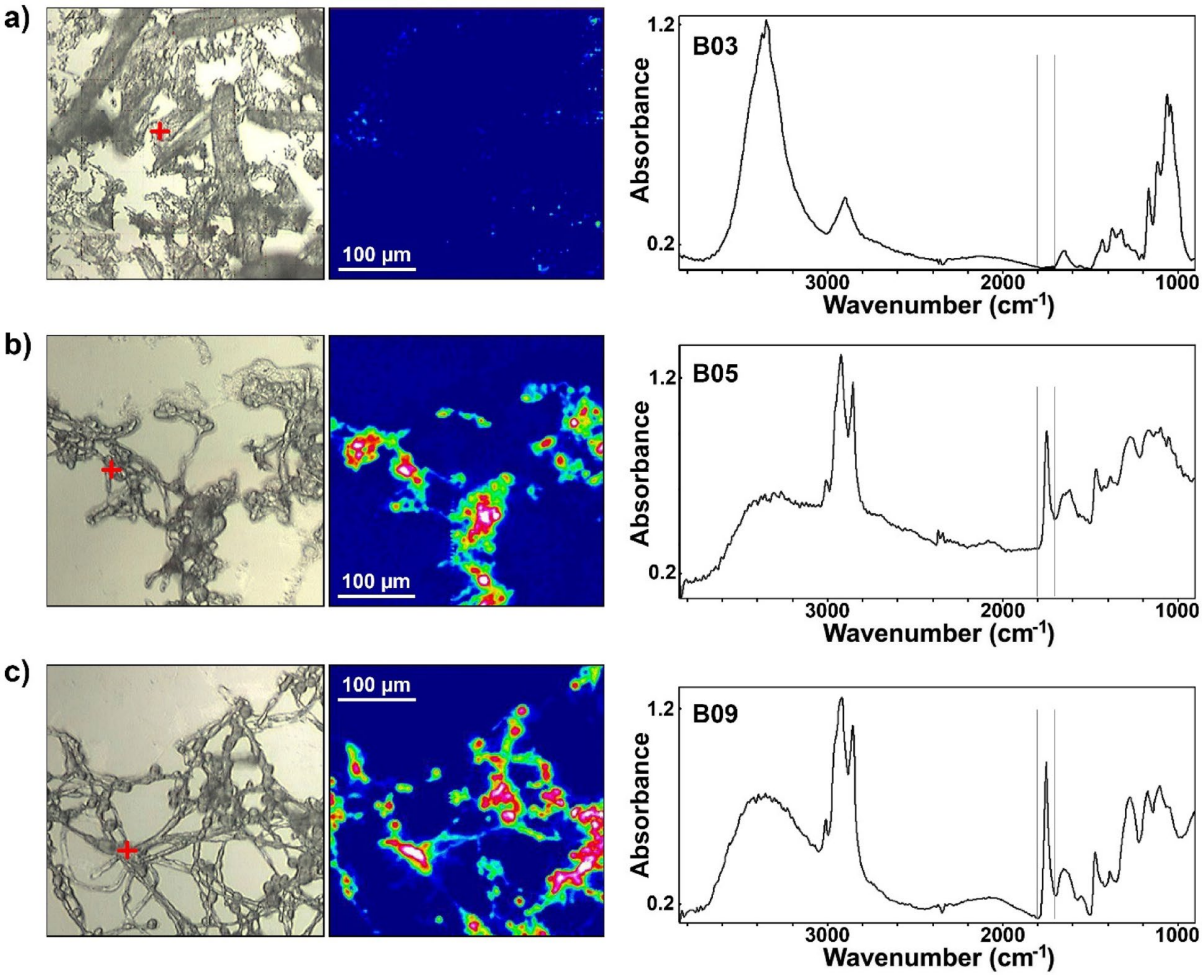
FTIR microspectroscopy of biomass at 216 h from: **a** cellulose-rich pulp control media (bioreactor B03), **b** SSF (bioreactor B05) and **c** SHF hydrolysate H2 (B09). Left column: visible microscopy image; middle column: FTIR microspectroscopy image created by integrating the spectral region 1800–1700 cm^− 1^; right column: spectra at positions marked with crosses in visible images (spectral region 1800–1700 cm^− 1^ is designated with lines)

FTIR microspectroscopy is a fairly simple method for at-line monitoring of the SSF process at a microscopic scale. As can be seen in Fig. 8, the chemical signals of cellulose-rich pulp substrate and fungal biomass are extremely different. Fungal biomass is characterised by the amide I band (related to –C=O stretching) at approx. 1640 cm^−1^ and amide II band (related to C–N–H deformation) at approx. 1545 cm^−1^. Both of these bands are related to the main building blocks of filamentous fungi: proteins, chitin, and chitosan [2, 11, 13]. Accumulation of SCOs in the fungal biomass results in a sharp increase of the lipid-related signal at 1745 cm^−1^ (–C=O stretching in esters) (Fig. 4b, c) [2, 11, 13]. Since the bands at 1745 and 1545 cm^−1^ are not present in the spectra of cellulose biomass (Fig. 4a), monitoring SSF process is straightforward by using FTIR microspectroscopy. Therefore, FTIR microspectroscopy can detect the conversion of cellulose-rich pulp substrate to fungal biomass and provide spatially resolved chemical information, enabling the visualization of lipid accumulation within the filamentous fungus *Mucor circinelloides*.

This demonstrates that FTIR microspectroscopy is a powerful tool for lab-scale fermentation process development and optimization, and, in general, for studying the bioconversion of lignocellulosic biomass into fungal biomass and metabolites. Traditional genetic and wet chemistry methods struggle in such complex systems, and while FTIR offers qualitative insights, its quantitative capabilities are limited. Based on our experience with developing quantitative regression models for lipids and other fungal metabolites [10], combined with recent advances in hyperspectral data preprocessing [39, 44], we believe that obtaining quantitative data from FTIR microspectroscopy images is now achievable. Recently, we demonstrated a deep learning-based calibration transfer method that adapts regression models developed for macroscopic infrared spectra to microscopic pixel spectra from hyperspectral IR images [73]. By mapping microspectroscopic FTIR data to the domain of bulk measurements, this approach enables quantitative chemical analysis in imaging applications. The method was validated using data from oleaginous filamentous fungi, and we believe it should also be effective for complex mixtures of lignocellulose substrate and fungal biomass. This approach could significantly enhance the utility of FTIR microspectroscopy for studying biomass conversion and lipid production, bridging the gap between qualitative and quantitative analysis.

## Conclusions

FTIR and FT-Raman spectroscopies are ideal for at-line monitoring of bioprocesses since they provide information on the chemical composition of both growth media and biomass. Monitoring SHF processes is very similar to monitoring standard processes with a single carbon source (as simulated here with glucose control media), and the same approach is effective in both cases. For example, as demonstrated in this study, FTIR with fiber optics (FTIR-ATR) is effective for monitoring glucose in SHF processes. Unfortunately, the traditional vibrational spectroscopy approach of monitoring growth media is of limited value for monitoring SSF processes by oleaginous filamentous fungi, due to relatively low concentrations of dissolved fermentable monosaccharides. For example, the accuracy of FTIR-ATR approach is limited in SSF processes because of the very low glucose concentrations. However, both FTIR-HTS and FT-Raman-HTS were able to measure changes in biomass composition, from cellulose-rich pulp substrate to lipid-rich fungal biomass. In particular, FT-Raman spectroscopy can obtain all the necessary information directly from cell suspension measurements (i.e., bulk raw bioreactor content), without any sample pre-treatment, purification, or modification. The results show that FT-Raman spectroscopy of cell suspension provides rich information on the chemistry of both lignocellulose substrate and fungal biomass, including lipid accumulation by oleaginous filamentous fungi. Thus, we have demonstrated that FT-Raman-HTS can be used as a rapid and effective at-line PAT tool for monitoring SSF processes for intracellular SCO production by filamentous fungi. This represents a remarkable simplification of the monitoring of complex SSF cultures. Notably, with a slight configuration adjustment for online monitoring, the same instrument enables faster spectral acquisition due to improved signal-to-noise ratio, highlighting FT-Raman spectroscopy as an ideal PAT tool for both online and at-line monitoring of SSF processes for intracellular SCO production. In addition, the study demonstrates the clear potential of FTIR microspectroscopy as a powerful tool for lab-scale SSF process development and optimization.

## Supplementary Information

Supplementary Material 1: The supplementary file includes supplementary details on biomass analyses, morphology, and data analyses.

## Data Availability

All datasets generated for this study are available in the Zenodo repository upon reasonable request from the corresponding author: https://doi.org/10.5281/zenodo.15181699.
