# Supplementary material for: Application of vibrational spectroscopies as process analytical techniques for monitoring fermentation and the conversion of lignocellulosic biomass by oleaginous filamentous fungi
Source: Microb Cell Fact. 2025 Dec 9;24:245. doi: 10.1186/s12934-025-02868-w (PMC12690941; doi:10.1186/s12934-025-02868-w)

Supplementary Material

Application of vibrational spectroscopies as process analytical techniques for monitoring fermentation and the conversion of lignocellulosic biomass by oleaginous filamentous fungi

Simona Dzurendova[1,2] (simona.dzurendova@fch.vut.cz), Cristian Bolaño Losada[1] (cristian.bolano.losada@nmbu.no), Benjamin Xavier Dupuy—Galet[1] (benjamin.dupuy.galet@nmbu.no), Ondrej Slany (ondrej.slany@nmbu.no), Kai Fjær[1] (kai.fjar@gmail.com), Francesca Di Bartolomeo[3] (Francesca.dibartolomeo@sintef.no), Sidsel Markussen[3] (Sidsel.Markussen@sintef.no), Alexander Wentzel[3] (Alexander.Wentzel@sintef.no), Anikó Várnai[4] (aniko.varnai@nmbu.no), Line Degn Hansen[4] (line.degn.hansen@nmbu.no), Svein Jarle Horn[4] (svein.horn@nmbu.no), Achim Kohler[1] (achim.kohler@nmbu.no), Volha Shapaval[1] (volha.shapaval@nmbu.no), Boris Zimmermann[1]* (boris.zimmermann@nmbu.no)

[1]Faculty of Science and Technology, Norwegian University of Life Sciences, Postbox 5003, 1432 Ås, Norway
[2]Faculty of Chemistry, Brno University of Technology, Purkyňova 464/118, 61200 Brno, Czech Republic
[3]SINTEF Industry, Postbox 4760 Torgarden, 7465 Trondheim, Norway
[4]Faculty of Chemistry, Biotechnology and Food Science, Norwegian University of Life Sciences, Postbox 5003, 1432 Ås, Norway

*Corresponding author:
Boris Zimmermann
Faculty of Science and Technology
Norwegian University of Life Sciences
Drøbakveien 31, 1432 Ås, Norway.
Tel: +47 6723 1576
Fax: +47 6496 5001
E-mail: boris.zimmermann@nmbu.no

Table of Contents **Page**

Figure S1. Schematic illustration of the overall research methodology S-2
Table S1. Composition of cellulose pulp and derived hydrolysates S-3
Figure S2. Visible microscopy image of biomass S-4
Table S2 Statistical analysis S-5
Figure S3. PLSR analysis of FTIR-ATR data S-6

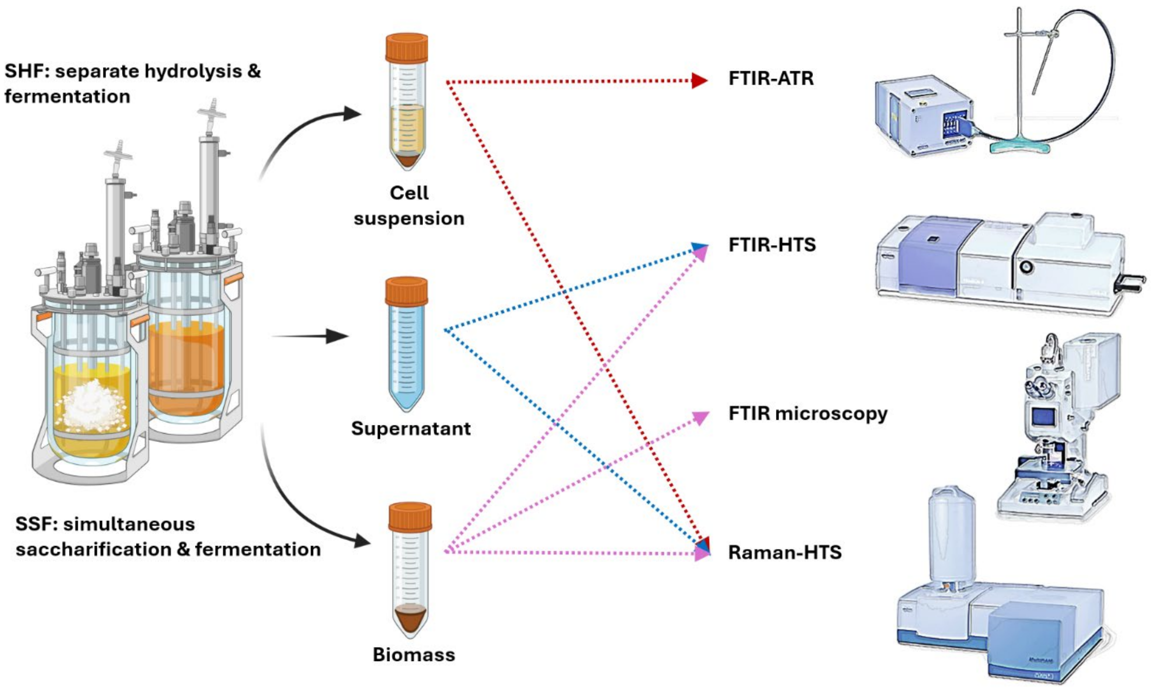

Figure S1. Schematic illustration of the overall research methodology.

Table S1. The composition of the Norway spruce pretreated pulp and derived hydrolysates used in this study. Data retrieved from Costa et al. [69] and Bolaño-Losada et al. [50] with the permission of the authors.

Component	Cellulose-rich BALI™ pulp [69]	Excello-90 [50]	NMBU BALI™ hydrolysate [50]
Glucose	87.4%	492.4 g/L	93.4 g/L
Xylose	2.7%	51.7 g/L	5.1 g/L
Arabinose	nd	4.9 g/L	na
Galactose	nd	4.9 g/L	na
Mannose	5.2%	44.4 g/L	3.5 g/L
Cellobiose	na	20.6 g/L	na
Gentiobiose	na	24.7 g/L	na
Fructose	na	14.0 g/L	na
Glycerol	na	12.3 g/L	na
Acetic acid	na	21 g/L	2.3 g/L
Lignin	3.3%	na	na
p-Coumaric acid	na	10.3 µg/g	na
Ferulic acid	na	35.2 µg/g	na
5-Hydroxymethylfurfural	na	23.3 ppm	na
Furfural	na	24.7 ppm	na
Ca^{2+}	na	0.183% (w/w)	na
Mn^{3+}	na	12.8 mg/kg	na
Fe^{2+}	na	9.04 mg/kg	na
Nitrogen	na	0.07 g/100 g	0.01g/100g

na: not analyzed

nd: not detected

[50] Losada CB, Di Bartolomeo F, Wentzel A, Markussen S, Dzurendova S, Zimmermann B, Fjaer K, Slany O, Várnai A, Hansen LD, et al: Simultaneous production of fatty acids and amino polysaccharides from Norway spruce hydrolysates using oleaginous Mucor circinelloides. Scientific Reports 2025, 15:14106.

[69] Costa THF, Kadic A, Chylenski P, Varnai A, Bengtsson O, Liden G, Eijsink VGH, Horn SJ: Demonstration-scale enzymatic saccharification of sulfite-pulped spruce with addition of hydrogen peroxide for LPMO activation. Biofuels Bioproducts & Biorefining-Biofpr 2020, 14:734-745.

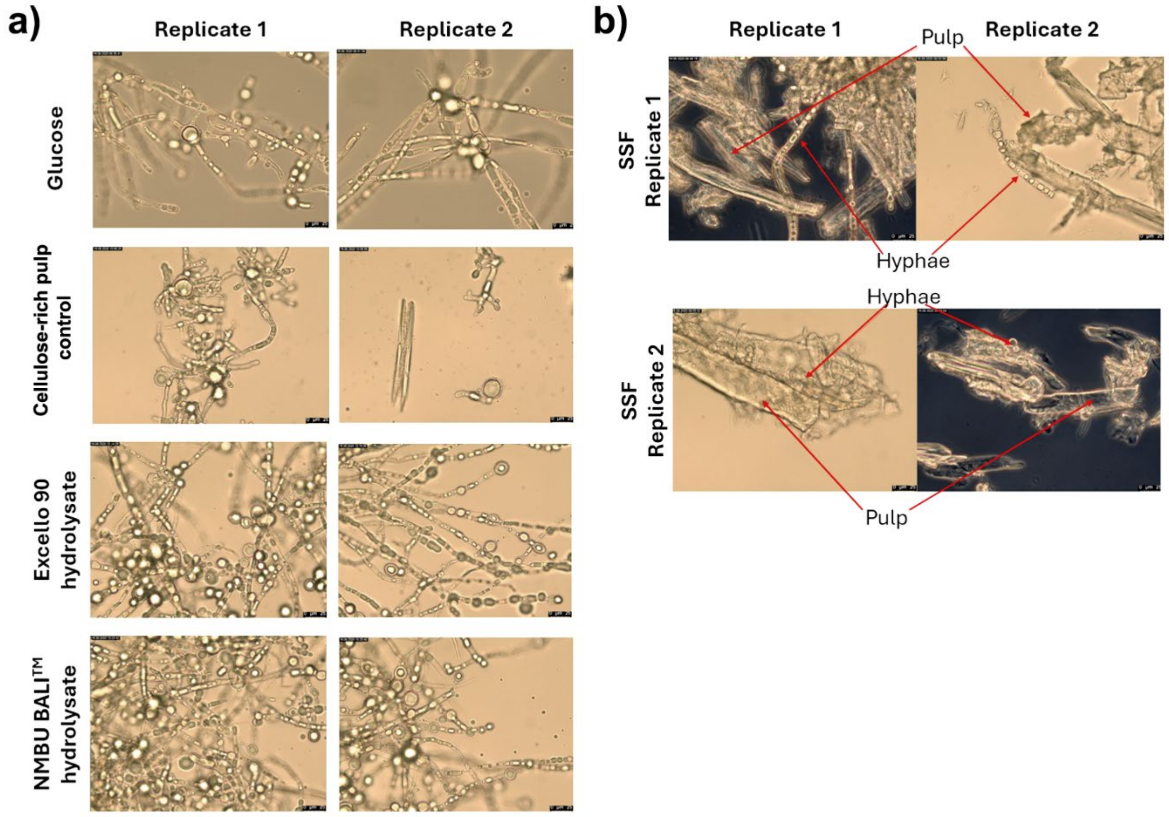

Figure S2. Visible microscopy image of biomass at 216h from A) B01-B02: glucose control; B03-B04: cellulose-rich pulp control; B07-B08: SHF hydrolysate H1; B09-B10: SHF hydrolysate H2, and B) B05-B06: SSF. Note the red arrows indicating the pulp fibers and fungal cells. Scale bar = 25 µm.

Table S2. Statistical analysis for total lipid content (as percentage of dry biomass, $\%_{w/w}$) and for total biomass concentration. ANOVA was employed to evaluate differences between: a) five different processes, and b) processes with SHF hydrolysate H1, SHF hydrolysate H2, and glucose control. Student's t-test was employed to evaluate differences between the two independent biological replicates. (* p<0.05, ** p<0.01, *** p<0.001; The symbol "×" designates no statistical significance.).

	ANOVA		Student's t-test				
	Whole dataset	SHF1, SHF2 & Glucose control	Glucose control	Cellulose-rich pulp control	SSF	SHF1	SHF2
Total lipids	***	×	×	×	×	×	×
Total biomass	***	×	×	×	×	×	×

Figure S3. PLSR analysis of FTIR-ATR data for cell suspension for estimation of glucose. a) The corresponding PLSR model and b) regression coefficients.

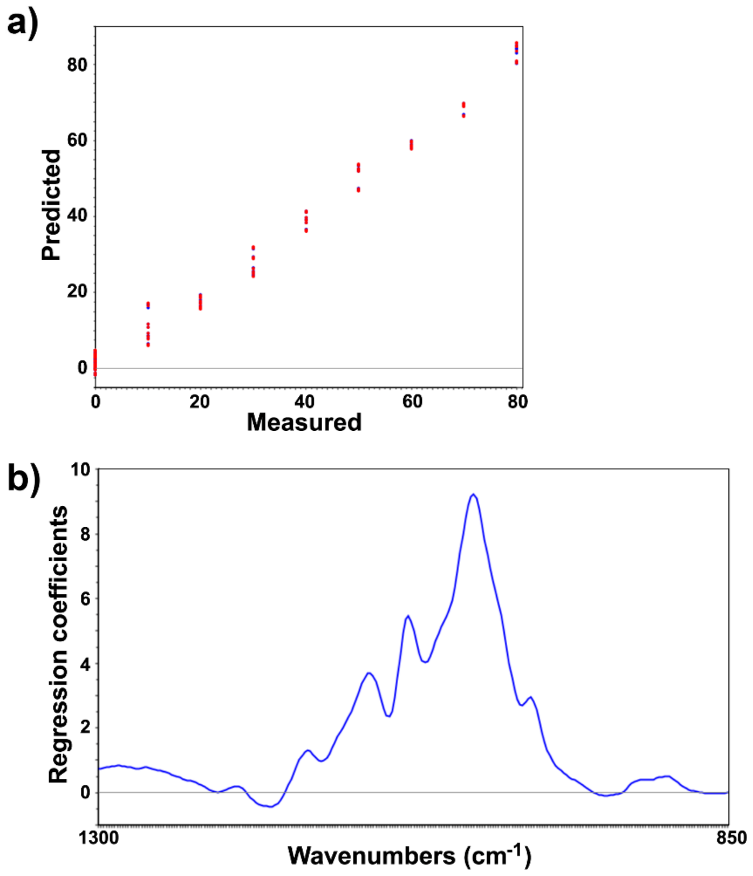

Supplement: Supplementary file 1 — Supplementary Material 1: The supplementary file includes supplementary details on biomass analyses, morphology, and data analyses. [file 12934_2025_2868_MOESM1_ESM.pdf]
